# 2023 FIGO Staging of Endometrial Cancer with Molecular Classification: Dawn and Challenges

**DOI:** 10.7150/jca.120459

**Published:** 2025-11-03

**Authors:** Wei Zhao, Qiqi Xu, Peiyu Feng, Dongxiao Hu, Hongyan Xu, Xiaofei Zhang, Wanrun Lin, Feng Zhou, Yang Li

**Affiliations:** 1 Department of Gynecologic Oncology, Women's Hospital, Zhejiang University School of Medicine, Hangzhou, Zhejiang, China.; 2 Zhejiang Provincial Clinical Research Center for Obstetrics and Gynecology, Hangzhou, Zhejiang, China.; 3 Department of Operating Room, Women's Hospital, Zhejiang University School of Medicine, Hangzhou, Zhejiang, China.; 4 Department of Pathology, Women's Hospital, Zhejiang University School of Medicine, Hangzhou, Zhejiang, China.; 5 Laboratory of Pathology, National Cancer Institute, National Institutes of Health, Bethesda, MD, USA.; 6 Departments of Pathology, The International Peace Maternal and Child Health Hospital, School of Medicine, Shanghai Jiao Tong University, Shanghai, China.; 7 Shanghai Key Laboratory of Embryo Original Diseases, Shanghai, China.; 8 Zhejiang Key Laboratory of Maternal and Infant Health, Hangzhou, Zhejiang, China.; 9 Traditional Chinese Medicine for Reproductive Health Key Laboratory of Zhejiang Province, China.

**Keywords:** Endometrial Cancer, Staging, Endometrial Cancer, Molecular Classification

## Abstract

**Objective:** To assess the prognostic performance of the 2023 FIGO staging system for endometrial cancer, which incorporates molecular classification (FIGO 2023m), we analyzed survival outcomes and compared them with the 2009 FIGO system (FIGO 2009).

**Methods:** We retrospectively reviewed 720 patients with endometrial cancer treated between 2013 and 2021. Staging was performed according to FIGO 2009 and FIGO 2023m. Progression-free survival (PFS) and overall survival (OS) were estimated using Kaplan-Meier analysis. Factors associated with survival were identified through univariate and multivariate Cox proportional hazards analyses.

**Results:** Of the 720 patients, 27.4% (197/720) were reclassified under FIGO 2023m, and 182 were upstaged from stage I to stage II, primarily due to p53 abnormalities (54.9%). Patients with stage I disease according to FIGO 2023m had comparable survival rates (PFS: 95.3% vs. 92.8%; OS: 99.2% vs. 95.9% under FIGO 2009). Within stage II, OS in patients classified as FIGO 2023m IIC was slightly lower than in stage IIC but did not differ statistically (92.3% vs. 86.9%). Aggressive histology, positive peritoneal cytology, and deep myometrial invasion were associated with poorer outcomes. Patients harboring POLE mutations showed excellent prognosis (5-year OS, 100.0%), even at advanced stages.

**Conclusion**: Compared with FIGO 2009, the FIGO 2023m staging system offers improved prognostic value and better discriminative ability. Incorporating molecular subtyping is crucial even in advanced disease. However, omitting peritoneal cytology from prognostic assessment may risk undertreatment. Continued refinement in quantifying lympho-vascular space invasion (LVSI) and differentiating complex endometrial-myometrial junctions from genuine myometrial invasion remains a challenge.

## Introduction

A precise staging system is essential for effective cancer management, guiding prognostication, treatment decisions, and comparative outcomes analysis. In 2023, the International Federation of Gynecology and Obstetrics (FIGO) revised the staging criteria for endometrial cancer (EC) 1. These revisions aim to improve the stratification of prognostic subgroups and provide clinically actionable subcategories. Notably, the main modifications of the FIGO 2023 staging system specify: (1) recognition of distinct histologic subtypes (aggressive vs. non-aggressive); (2) categorization of lymphovascular space invasion (LVSI) as absent, focal, or substantial; (3) differentiation of adnexal involvement; and (4) distinction between micro- and macro-metastases in lymph nodes.

Progress in characterizing EC at the molecular level has also shaped diagnostic and therapeutic strategies. The Cancer Genome Atlas (TCGA) classifies EC into four genomic subgroups 2: POLE ultra-mutated (POLEmut), microsatellite instability-high or mismatch repair deficient (MMRd), somatic copy number low (NSMP), and somatic copy number high (p53abn). These molecular subtypes have distinct prognoses, with POLEmut tumors conferring the most favorable outcome, p53abn tumors the least, and MMRd/NSMP tumors an intermediate outlook 3-5.

Despite these advances, the revised FIGO staging system requires further validation. This study evaluates the prognostic impact of a 2023 staging approach integrating molecular profiling compared with the established FIGO 2009 system. We aim to determine whether the new FIGO 2023m system more accurately reflects patient outcomes.

## Materials and Methods

### Case selection

We retrospectively analyzed 720 patients with EC treated at the Women's Hospital of Zhejiang University from January 2013 to December 2021. We collected clinical and pathological data, including age, body mass index (BMI), peritoneal cytology, surgical approach, tumor histology, and molecular subtype. Patients who had undergone their operations outside of our hospital or those lack follow-up information after surgery were excluded. The Institutional Review Board of our center approved this study.

All the hematoxylin and eosin (H&E)-stained and immunohistochemistry slides were reviewed by senior gynecologic pathologists. All tumors were classified according to the 2020 WHO classification of female genital tumors 6. LVSI was evaluated and classified as negative, focal (< 5 vessels involved), or extensive (≥ 5 vessels involved) 1. For tumors demonstrating multiple molecular features (e.g., POLEmut or MMRd coexisting with secondary p53abn), classification favored POLEmut or MMRd to reflect the more favorable prognosis.

All patients were staged using both FIGO 2009 and FIGO 2023m. “Upstaging” refers to reclassification to a more advanced category, and “downstaging” refers to assignment to a less advanced category.

### Molecular classification

Tumor samples were classified into four molecular subtypes (POLEmut, MMRd, p53abn, or NSMP) according to WHO-endorsed criteria 2. DNA extracted from five consecutive 10-μm FFPE sections using the NuClean FFPE DNA Kit (CW 2646, China) was analyzed for POLE mutations via a custom PCR assay (Dalton-MIT™) targeting nine hotspot sites in exons 9 - 14 7. MMRd was defined by loss of nuclear staining (vs. internal controls) for ≥ 1 mismatch repair protein (MLH1, MSH2, MSH6, or PMS2) on IHC, while p53abn required either complete nuclear loss (with intact internal control), strong nuclear overexpression (> 80% tumor cells), cytoplasmic staining, or subclonal mutant expression (≥ 5% tumor cells with mixed patterns) 4. Tumors negative for POLEmut, MMRd, and p53abn were classified as NSMP.

### Analysis

The IBM SPSS Statistics 29.0 software was utilized to perform the statistical analysis. Descriptive statistics were employed to portray the demographic characteristics of the patients. The survival curve was visualized using the Kaplan-Meier method. To evaluate the significance of individual covariates on survival time, the Cox proportional hazards model was used. A *P-*value below 0.05 was regarded as statistically significant.

## Results

### Patient characteristics and outcomes

**Table 1** presents the clinical and pathological features of the 720 patients. Their median age was 56 years (IQR 51-62), and the median BMI was 24.0 kg/m² (IQR 22.0-26.7). Endometrioid EC was the most common histopathological type (553 patients, 76.8%), while non-endometrioid EC accounted for 23.2%. Myometrial invasion was observed in 688 patients (95.6%), and 163 (22.6%) had deep myometrial invasion. In terms of surgical approach, 409 patients underwent laparoscopic surgery and 311 underwent laparotomy.

### Transition from FIGO 2009 to FIGO 2023m

**Figure 1:**
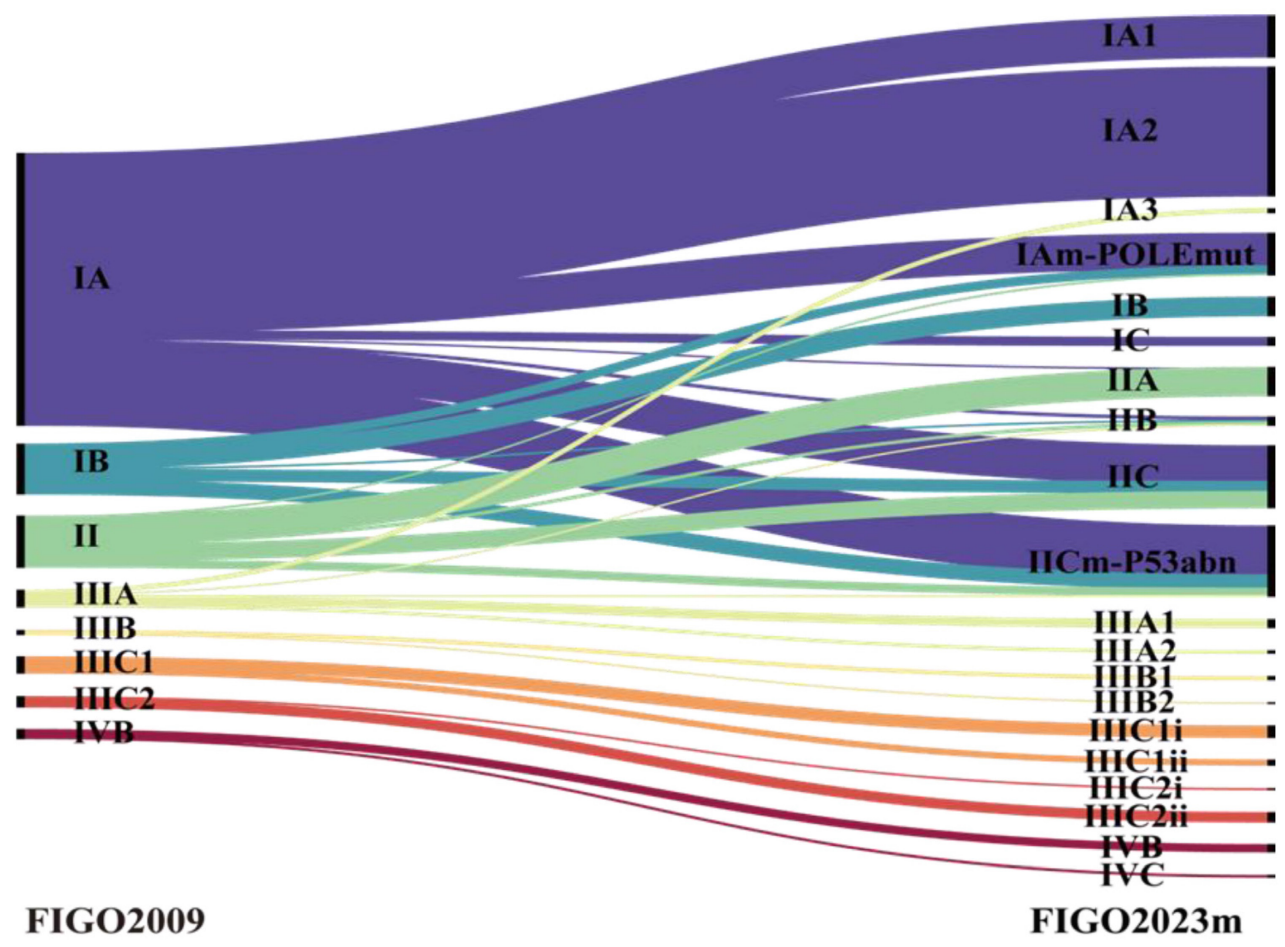
Transition of (sub)stages from FIGO 2009 to FIGO 2023. Results of main stages are written in bold letters. POLEmut, POLE mutated; p53abn, p53 abnormal.

**Table 2** shows the distribution of disease stages under both FIGO 2009 and FIGO 2023m. According to FIGO 2009, 625 patients (86.8%) had early-stage disease (stage I or II), 80 (11.1%) were in stage III, and 15 (2.1%) were in stage IV. **Figure 1** illustrates how patients were reallocated under FIGO 2023m, indicating that 27.4% of them changed stage. Of these, 182 (25.3%) moved from stage I to stage II, primarily due to abnormal p53 (54.9%), invasive histology (40.7%), or substantial LVSI (4.4%). Thirteen patients (1.8%) were downstaged, including 3 from stage II to IAm-POLE, 6 from stage IIIA1 to IA3, and 4 from stage IIIA1 to IICm-p53 (IA3 with p53abn).

### Molecular subtypes

Molecular subtypes were identified in 700 (97.2%) patients, with the remaining 2.8% unclassifiable because of insufficient tissue samples. The POLEmut subgroup comprised 74 (10.6%) patients, the MMRd subgroup 161 (23.0%), the NSMP subgroup 314 (44.9%), and the p53abn subgroup 151 (21.6%) (**Supplemental Table 1**).

**Figure 2:**
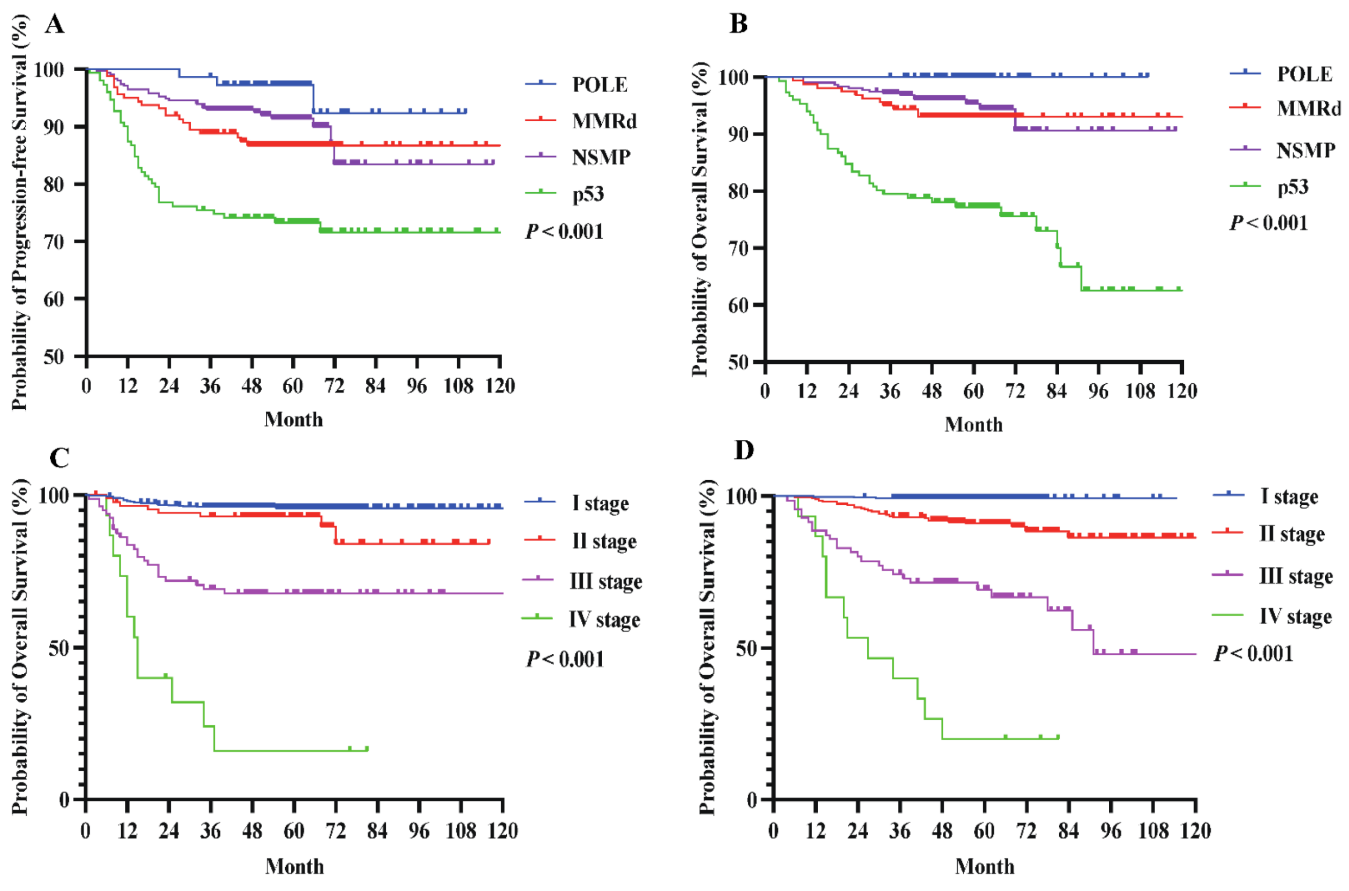
The 5-year (A) PFS and (B) OS rates for molecular subtypes among EC patients. The OS curves for patients with EC stage I-IV according to (A) FIGO 2009, (B) FIGO 2023m. P value of <0.05 was considered significant.

During a median follow-up of 55 months (IQR 47-66) for PFS and 56 months (IQR 47-67) for OS, 92 (12.8%) patients developed tumor recurrence, and 65 (9.0%) died of disease progression. As shown in **Figure 2**, patients with POLE mutations had excellent outcomes, with a 100% 5-year OS rate, while those with p53 abnormalities had the poorest 5-year PFS (72.8%) and OS (74.2%). Individuals classified as MMRd or NSMP had intermediate survival rates.

### Prognostic impact of the 2023 FIGOm staging system

**Figure 3:**
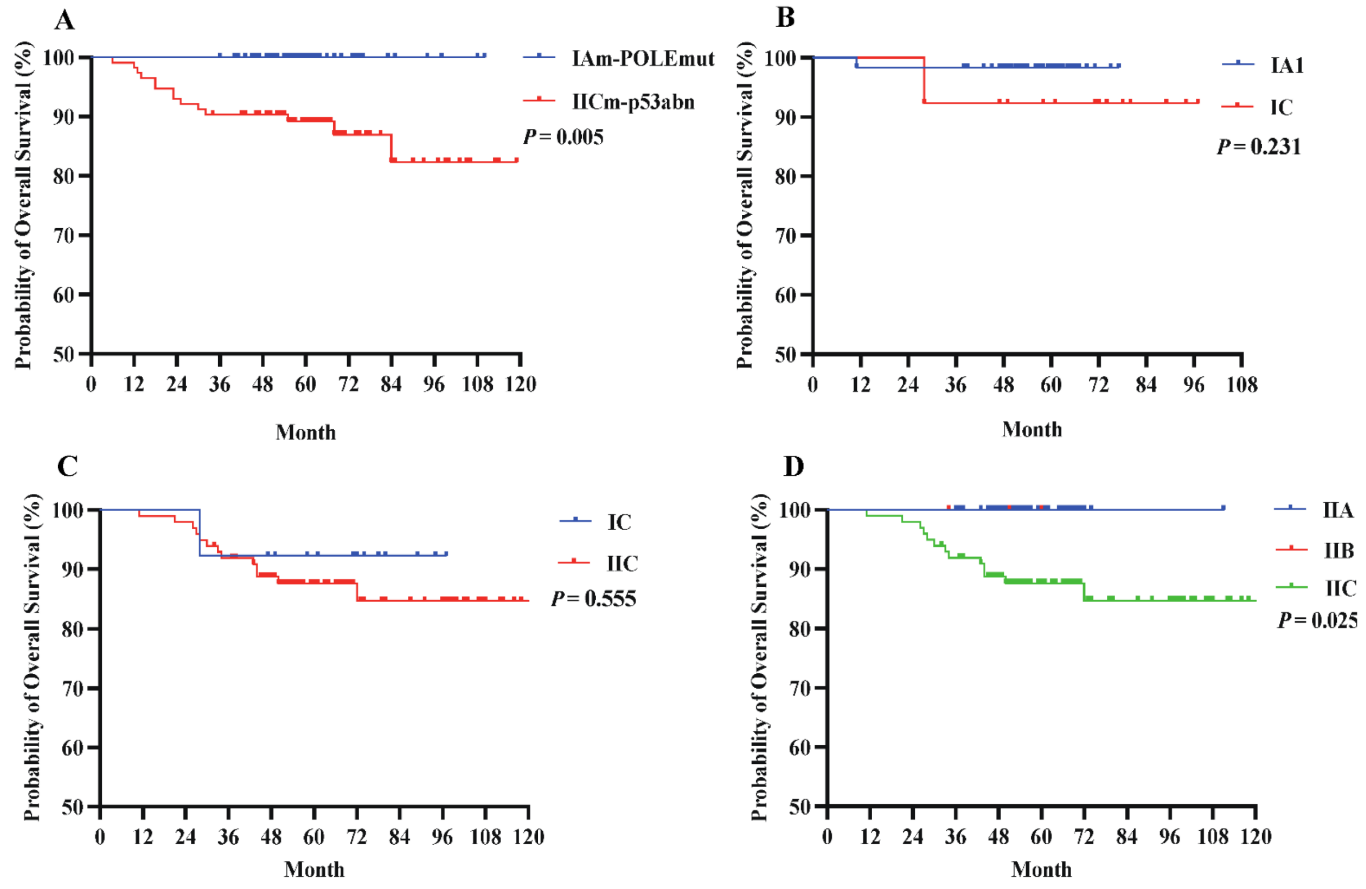
(A) OS compared by the early stage in the FIGO 2023m system. (B) OS compared by the histological types at early stages in the FIGO 2023m system. (C) OS compared by the myometrial invasion in the FIGO 2023m system. (D) OS compared by the LVSI status in the FIGO 2023m system.

**Table 3** outlines the 5-year PFS and OS rates by (sub)stage for both staging systems. Among stage I patients, those categorized using FIGO 2023m achieved comparable PFS (95.3% vs. 92.8%) and OS (99.2% vs. 95.9%) compared with FIGO 2009. Stage II disease showed similar outcomes under both versions (PFS: 86.8% vs. 86.2%; OS: 90.1% vs. 90.8%). In contrast, stage III patients classified by FIGO 2023m had lower PFS (57.1% vs. 60.0%) and OS (64.3% vs. 68.8%) than those categorized by FIGO 2009, whereas stage IV outcomes did not differ between the two systems (Figure 3).

Under FIGO 2023m, early-stage EC generally exhibited a favorable prognosis. Notably, patients with POLEmut had no deaths during the study period, and those with stage IAm-POLEmut had a 100% OS rate. Conversely, patients with stage IICm-p53abn had a poorer OS (87.7%; Table 4), significantly different from that of the POLEmut cohort (P = 0.005, **Figure 3A**). When tumors were limited to endometrial polyps or the endometrium, outcomes remained excellent regardless of aggressive histology (stage IC) or not (stage IA1), and the OS difference was not statistically significant (92.3% vs. 98.4%, P = 0.231, **Figure 3B**). A slight trend emerged favoring higher OS for stage IC compared to stage IIC (92.3% vs. 86.9%, **Figure 3C**), though this difference was not significant (P = 0.555), likely owing to limited sample size. Importantly, no significant OS differences were seen between stage IIB and IIA, but patients at stage IIA or IIB fared better than those at stage IIC (P = 0.025, **Figure 3D**).

**Figure 4:**
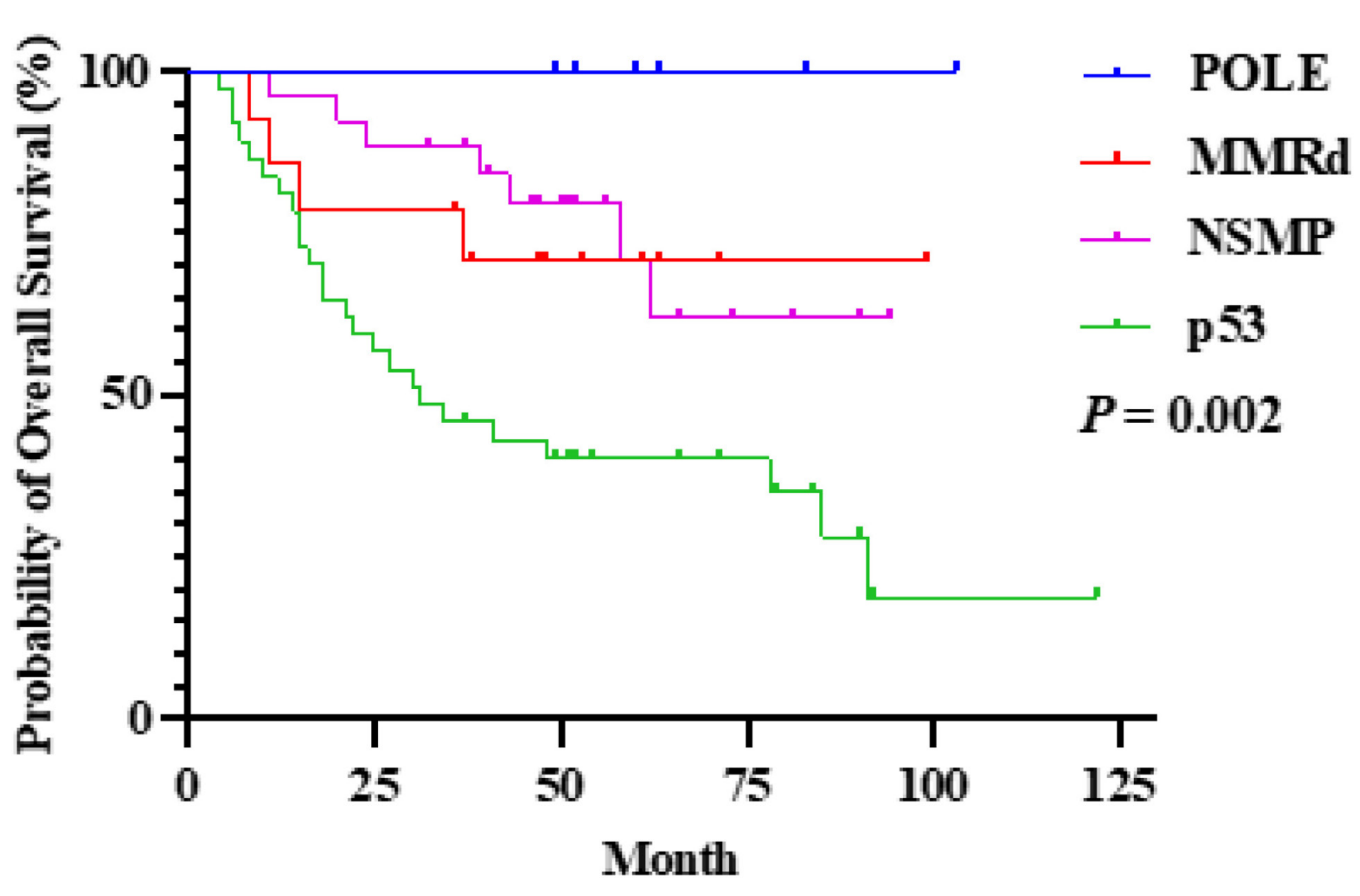
Overall survival rate compared by the molecular subtypes at stage III and IV in the FIGO 2023m system.

Patients with *POLE*mut had a good prognosis, even at advance stages including stage III or IV, with 5-year OS rate of 100%. The 5-year OS rate of patients with *POLE*mut was significantly better than those with the p53 abnormalities (100.0% versus 32.4%, *P* = 0.002) (**Supplemental Table 2, Figure 4**).

To evaluate the impact of histological types, peritoneal washing cytology, myometrial involvement, surgical approach, and lymphovascular invasion on OS rates, the univariate and multivariate analysis using a Cox proportional hazards model were performed. Aggressive histological types had a 7.3-fold increased risk of mortality compared to those with non-aggressive types (*P* < 0.001, **Table 4**). Positive peritoneal cytology (PPC) remained a significant predictor of survival even after balancing other contributing factors (*P* < 0.001, **Table 4**). Patients with PPC had a 6.2-fold increased risk of mortality compared to those with negative peritoneal cytology (NPC). Patients with superficial myometrial invasion or no myometrial invasion did not differ significantly, while patients with deep myometrial invasion had a 4.8-fold increased risk of mortality compared to those with no myometrial invasion (*P* = 0.012, **Table 4**). Furthermore, no significant difference in mortality risk were found based on surgical approach or the presence of LVSI involvement (*P* > 0.05).

## Discussion

In this study, we evaluated the discriminatory ability of FIGO 2009 versus FIGO 2023m for predicting outcomes in endometrial cancer (EC). We found that 25.6% of patients were upstaged, primarily because of p53 abnormalities and aggressive tumor histology. Notably, stage I patients under FIGO 2023m had comparable 5-year PFS and OS rates compared with those staged under FIGO 2009, whereas stage III patients under FIGO 2023m had lower rates than those staged under FIGO 2009. The main reasons are that: (1) the new FIGO 2023m substages IAm-POLEmut and IICm-p53abn reflected highly favorable or poor outcomes, respectively; and (2) some patients were downstaged from IIIA to IA3, which improved the prognostic precision of the staging system. Overall, FIGO 2023m demonstrates better stage differentiation and higher predictive accuracy for OS compared with FIGO 2009, particularly in early disease. This shift indicates that FIGO 2023m is more adept at identifying early-stage patients with better long-term survival, while also applying more stringent criteria for stage III.

Several comparative studies have assessed the performance of FIGO 2009 and FIGO 2023. For example, Schwameis reported that most patients were upstaged due to classifying aggressive types of myometrial invasion as stage IIC 8. Matsuo emphasized the relevance of distinguishing micro- from macro-metastatic lymph nodes 9. Meanwhile, Mayumi Kobayashi-Kato et al. revealed that incorporating molecular classification into FIGO2023 improved prognosis stratification more than either FIGO 2009 or FIGO 2023 alone 10. Consistent with these findings, our data showed excellent outcomes for patients with POLE-mutated tumors and the poorest outcomes for those with p53 abnormalities.

An interesting observation was that when the tumor remained confined to the endometrium (i.e., no myometrial invasion), both FIGO 2023m IA and IC demonstrated high OS rates, regardless of aggressiveness. This is in line with other studies indicating that serous EC confined to the uterus can show a relatively favorable prognosis 11, 12. However, it is also important to recognize that many presumed localized cases can harbor subclinical extrauterine disease 13. To address this, FIGO 2023m employs more vigilant categorization for aggressive disease, even when it appears confined.

Substantial LVSI has been identified as a useful prognostic indicator associated with poor outcomes in EC 14. Yet, reproducibility in quantifying LVSI remains a challenge because no universally accepted standard exists for whether to measure the maximum involvement on a single slide or the cumulative extent across multiple slides 15-17. Interestingly, we did not find a worse prognosis for patients at stage IIB compared to IIA, but this may reflect the small number of IIB cases in our study.

The FIGO 2023m criteria for advanced-stage EC remain based on established surgical and clinicopathological features, but we observed that even at stages III and IV, POLEmut tumors still exhibited an outstanding 5-year OS rate of 100%. In the PORTEC-2 trial, POLEmut versus POLE wild-type EC had a 10-year recurrence-free survival of 100% vs. 80.1%, reinforcing that POLEmut tumors possess intrinsically favorable biology, irrespective of treatment 18-20. In contrast, p53 status is a well-known negative prognostic marker 21, 22, influencing outcomes even at the earliest stages, although more data are needed to confirm these observations in special cases.

Peritoneal cytology, while no longer part of FIGO staging criteria, remains reportable. Numerous studies have shown conflicting results regarding its prognostic significance in early-stage disease 23, but several large investigations have identified positive peritoneal cytology as an independent risk factor 24, 25. Our findings concur, showing a 6.2-fold higher death risk for patients with positive versus negative cytology. A Cox model adjusting for histological subtype and stage supported its prognostic value, in line with other reports 25-27. Therefore, excluding cytology from the staging algorithm might lead to undertreatment in certain subgroups.

We also observed a significantly poorer prognosis for patients with deep myometrial invasion compared to those without invasion, but no significant difference between superficial invasion and no invasion. Whether superficial invasion justifies upgrading in FIGO 2023m warrants additional research.

In summary, FIGO2023m offers superior prognostic accuracy for EC compared with FIGO2009, showing enhanced predictive ability and more precise stratification. Its main innovations involve downstaging early POLEmut cases to IAm-POLEmut and upstaging p53-abnormal cases to II Cm-p53abn. Moreover, molecular subtyping can be critical even at stages III and IV, as POLEmut remains a favorable predictor. Our results also emphasize that aggressive histological subtypes, deep myometrial invasion, and positive peritoneal cytology are associated with worse survival, suggesting that excluding cytology entirely from staging could lead to insufficient treatment for some patients.

## Supplementary Material

Supplementary tables.

## Figures and Tables

**Table 1:** Characteristics of patients with endometrial cancer (n=720).

Characteristic	Value
Age (year), (median, range)	56 (51- 62)
BMI (kg/m^2^), (median, range)	24.0 (22.0 - 26.7)
**Histological types, n (%)**	
Endometrioid cancer	553 (76.8)
Grade 1	276 (38.3)
Grade 2	201 (27.9)
Grade 3	76 (10.6)
Non-endometrioid cancer	167 (23.2)
**Peritoneal cytology, n (%)**	
Negative	696 (96.7)
Positive	24 (3.3)
**Myometrial involvement, n (%)**	
No myometrial invasion	32 (4.4)
Myometrial invasion less than 50%	525 (73.0)
Myometrial invasion of 50% or more	163 (22.6)
**Surgical approach, n (%)**	
Laparoscopic surgery	409 (56.8)
Laparotomy surgery	311 (43.2)
**Lymphovascular invasion, n (%)**	
Negative	605 (84.0)
Positive	115 (16.0)

**Table 2:** Distribution of each stage among the two staging systems.

	FIGO 2009 (n = 720)	Stage	FIGO 2023m (n = 720)
Stage I, n (%)	538 (74.7%)	Stage I, n (%)	363 (50.4%)
IA	453	IA1	61
		IA2	186
		IA3	7
		IAm-*POLE*mut	68
IB	84	IB	28
		IC	13
Stage III, n (%)	87 (12.1%)	Stage II, n (%)	272 (37.8%)
		IIA	44
		IIB	15
		IIC	99
		IICm-p53abn	114
Stage III, n (%)	80 (11.1%)	Stage III, n (%)	70 (9.7%)
IIIA	29	IIIA1	13
		IIIA2	5
IIIB	7	IIIB1	6
		IIIB2	1
IIIC1	27	IIIC1i	18
		IIIC1ii	9
IIIC2	18	IIIC2i	3
		IIIC2ii	15
Stage IV, n (%)	15 (2.1%)	Stage IV, n (%)	15 (2.1%)
IVB	15	IVB	12
		IVC	3

**Table 3:** 5-year PFS and OS rates in 722 EC patients according to the two staging systems.

Stage	FIGO 2009 (n=720)		Stage	FIGO 2023m (n=720)	
	PFS rate	OS rate		PFS rate	OS rate
Stage I	92.8%	95.9%	Stage I	95.3%	99.2%
IA	94.7%	97.8%	IA1	95.1%	98.4%
			IA2	96.2%	99.5%
			IA3	85.7%	100.0%
			IAm-*POLE*mut	95.6%	100.0%
IB	82.1%	85.7%	IB	96.4%	100.0%
			IC	84.6%	92.3%
Stage II	86.2%	90.8%	Stage II	86.8%	90.1%
			IIA	95.5%	100.0%
			IIB	80.0%	100.0%
			IIC	84.8%	86.9%
			IICm-p53abn	86.0%	87.7%
Stage III	60.0%	68.8%	Stage III	57.1%	64.3%
IIIA	69.0%	82.8%	IIIA1	69.2%	84.6%
			IIIA2	40.0%	40.0%
IIIB	71.4%	71.4%	IIIB1	66.7%	66.7%
			IIIB2	NS	NS
IIIC1	63.0%	74.1%	IIIC1i	77.8%	83.3%
			IIIC1ii	33.3%	55.6%
IIIC2	38.9%	38.9%	IIIC2i	NS	NS
			IIIC2ii	46.7%	46.7%
Stage IV	20.0%	20.0%	Stage IV	20.0%	20.0%
IVB	20.0%	20.0%	IVB	25.0%	25.0%
			IVC	0.0%	0.0%

**Table 4:** Cox multivariate survival analysis with histologic types, peritoneal washing cytology, myometrial invasion, surgical approach, and LVSI as prognostic factors.

Covariate		Hazard ratio (95% CI)	P value
Histological types			
	Non-aggressive	1	
	Aggressive	7.257 (3.389-15.539)	< 0.001
Peritoneal washing cytology			
	Negative	1	
	Positive	6.239 (3.258 - 11.950)	< 0.001
Myometrial involvement			
	No myometrial invasion	1	
	Superficial myometrial invasion	1.209 (0.357 - 4.100)	0.760
	Deep myometrial invasion	4.762 (1.415 - 16.033)	0.012
Surgical approach			
	Laparoscopic surgery	1	
	Laparotomy surgery	1.742 (0.884 - 3.433)	0.109
LVSI			
	Negative	1	
	Positive	0.911 (0.519-1.599)	0.744
