# Supplementary material for: 2023 FIGO Staging of Endometrial Cancer with Molecular Classification: Dawn and Challenges
Source: J Cancer. 2025 Nov 3;16(15):4400–7. doi: 10.7150/jca.120459 (PMC12664729; doi:10.7150/jca.120459)
Supplement: Supplementary file 1 — Supplementary tables. [file jcav16p4400s1.pdf]

Supplemental Table 1. The 5-year PFS and OS rates for molecular subtypes among EC patients.

Molecular subtype	*POLE*mut	MMRd	NSMP	p53abn
Sample size, n (%)	74 (10.6%)	161 (23.0%)	314 (44.9%)	151 (21.6%)
Number of recurrences, n	3	21	28	41
PFS rate (%)	95.9	87.0	91.1	72.8
Number of deaths, n	0	11	15	39
OS rate (%)	100.0	93.2	95.2	74.2

Supplemental Table 2. Overall survival rate compared by the molecular subtypes at stage III and IV in the FIGO 2023m system

Stage	*POLE*mut	MMRd	NSMP	p53abn	***P***
Sample size, n	6	14	26	37	
Number of deaths, n	0	4	8	25	
OS rate, %	100.0	71.4	69.2	32.4	0.002
